# Whole-genome sequence of the plant-associated bacterium *Pseudomonas granadensis* CT364 isolated in Seville, Spain

**DOI:** 10.1128/mra.00735-23

**Published:** 2024-04-30

**Authors:** Eva Cea-Torrescassana, Thomas P. Howard, Jem Stach, Maria del Carmen Montero-Calasanz

**Affiliations:** 1 School of Natural and Environmental Sciences, Newcastle University, Newcastle upon Tyne, United Kingdom; 2 IFAPA Las Torres- Andalusian Institute of Agricultural and Fisheries Research and Training, Junta de Andalucía, Cra. Sevilla-Cazalla, Alcalá del Río, Seville, Spain; University of Strathclyde, Glasgow, United Kingdom

**Keywords:** whole genome, plant growth promotion, biostimulant

## Abstract

*Pseudomonas* sp. CT364 was isolated from olive tree rhizosphere in Seville (Spain). We report its complete genome sequence, acquired by co-assembling Illumina and Nanopore reads. The genome comprises a circular chromosome of 6.2 Mbp and a G + C content of 60.0%. Taxonomic analyses confirmed it to be *Pseudomonas granadensis*.

## ANNOUNCEMENT

Strain CT364 promotes the growth of olive tree cuttings, induces root elongation in canola, and improves root development in mung beans and black-eye peas (1). Here, we report its complete genome.

CT364 was isolated in 2006 from the olive tree rhizosphere. Three soil rhizosphere samples collected near the roots of a single tree at 30 cm depth were combined, and homogenized in M79 salts (2), and 10-fold serial dilutions were plated on NFb agar aerobically at 28°C (3). A pure culture was obtained by streaking an isolated colony three times onto Tryptone Soy Agar (TSA) and incubating overnight at 28°C. Axenic cultures from single colonies were preserved at −80°C in 25% glycerol.

Genomic DNA was sequenced by long- and short-reads (Microbes NG (https://microbesng.com/) and short-reads (Novogene (https://www.novogene.com/). Pure bacterial biomass grown on a streaked Tryptone Soy Agar (TSA) plate overnight at 28°C was collected in cryopreservative tubes (Microbank, UK) and transported to MicrobesNG for DNA extraction by Wizard Genomic DNA Purification kit (Promega, USA). Short-read library was prepared using Nextera XT Library Preparation kit (Illumina, USA) and sequenced by NovaSeq6000 Illumina platform using a 250 bp paired-end protocol. Long-reads were barcoded by SQK-RBK004 and Native Barcoding by EXP-NBD104 (ONT, Oxford, UK), sequenced by GridION (ONT, UK) and basecalled by high-accuracy model using Guppy v.4.2.2 (4). For Novogene, a single colony of a streaked TSA plate grown overnight at 28°C obtained from the same frozen stock as that used for MicrobesNG was collected for genomic extraction by QIAGEN Genomic-tip 20/G kit (Venlo, Netherlands). The library was prepared by restriction enzyme fragmentation, end repair, and PCR amplification, followed by A-tailing and adapter ligation. A 150 bp paired-end sequencing was performed using Novaseq 6000 (Illumina, San Diego, USA). Both sequencing workflows are in Table 1.

**TABLE 1:** *Pseudomonas* granadensis CT364 whole-genome sequencing information and genomic statistics

Sequencing information	Number of reads	Total sequence length (Mbp)	Genome coverage	N50 (bp)
Illumina library 1 (SRX19847623), Novogene	2,005,338	422.68	∼68X	-
Illumina library 2 (SRX19847624), Microbes NG	43,210			
Nanopore (SRX19847625), Microbes NG	18,079	330.9	∼45X	34,392
Genome content information	Value			
Genome size (bp)	6,208,260			
Gene number	5,470			
GC content (%)	60.01			
CDSs (total)	5,373			
Genes (coding)	5,307			
Regulatory and miscellaneous features	85			
RNA genes	97			
rRNA: 5S, 16S, 23S	7, 6, 6			
tRNA	74			
noncoding RNA (ncRNA)	4			
Transfer-messenger RNA (tmRNA)	1			
Pseudogenes	66			

Short- and long-read quality was evaluated using FASTQC v.0.12.1 (5) and NanoStat v.1.6.0 (6), respectively. Adapters were removed using fastp v0.23.0 (7), and duplicate reads by NGSReadsTreatment v1.3 (8). A hybrid assembly combining both read types (including the three read sets) was performed using Unicycler v0.4.8 (9), which identified and closed the overlap and rotated the assembly to the starting gene *repA,* resulting in a single circularized contig. Quality control of the assembly was done by QUAST v4.0 (10) and genome annotation by Prokaryotic Genome Annotation Pipeline (PGAP) version 5.0 (11). Default parameters were used for all software.

**Fig 1:**
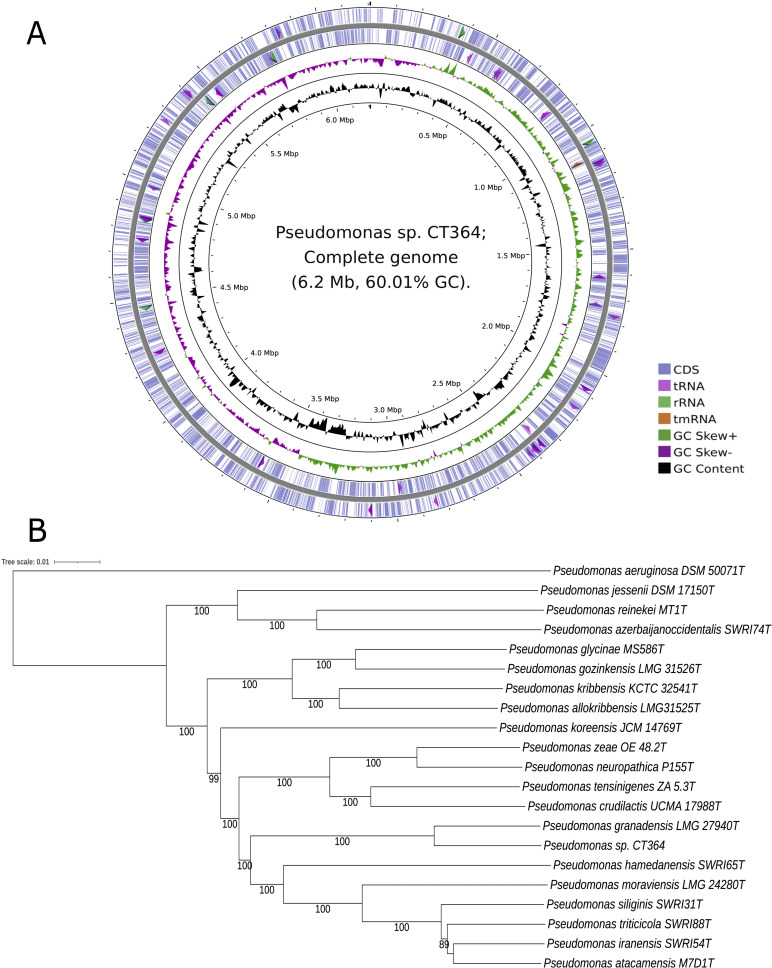
Genomic features and evolutionary relationships of *Pseudomonas granadensis* CT364. (**A**) Chromosome map of *P. granadensis* CT364. The coding sequence (CDS), rRNA, and rRNA are depicted in the external two circles. The third circle represents the GC skew curve (positive GC skew, green; negative GC skew, violet). The fourth circle shows the GC content (black). The map was generated by Proksee 2023 Server (17). (**B**) Whole-genome-based phylogenetic tree created with TYGS v389 (12) pipeline for *P. granadensis* CT364 and its 16S rDNA closely related sequenced *Pseudomonas* strains and *P. aeruginosa* DSM 50,071T as an outgroup. The length of the branches depicts the genetic distances. Tree inferred with FastME 2.0 (18) from GBDP distances calculated from genome sequences. The branch lengths are scaled in terms of the GBDP distance formula d5. The numbers above branches are GBDP bootstrap values >70%, with an average branch support of 97.3%. The three were rooted at the midpoint.

The genome comprises a circular chromosome of 6,208,260 bp, 5,470 genes (Fig. 1A), and the G + C content of 60.01% (Table 1). A whole-genome-based tree was built by TYGS v389 (12), including the 16S rRNA (1) closest genomes predicted by EzBioCloud v.2023.08.23 (13), and outgroup *Pseudomonas aeruginosa* DSM 50071^T^ (CP012001) (14) (Fig. 1B). The average nucleotide identity (ANI) was calculated by EzBioCloud and digital DNA-DNA hybridization (dDDH) by GGDC v2.1 (15). *Pseudomonas granadensis* LMG 27940^T^ (NZ_LT629778.1) was the closest relative of CT364 with ANI (95.5%) and dDDH (90.1%) values over the recognized species boundaries (95% and 70%) (15, 16). Accordingly, *P. sp*. CT364 belongs to the species *Pseudomonas granadensis*.

## Data Availability

The genome (CP069352) was deposited in INSDC (BioProject PRJNA695429 and BioSample SAMN17614312). The 16S rRNA sequence accession is EU336940. The Assembly accession is 66 GCA_016859165.1 and the raw sequence reads are SRX19847625, SRX19847624, and SRX19847623. The strain is deposited in the DSMZ culture collection (DSM 25356).
